# Crystal structure of 7,15-bis­(4-*tert*-butyl­phen­yl)-1,9-di­methyl­hepta­zethrene

**DOI:** 10.1107/S2056989016020247

**Published:** 2017-01-06

**Authors:** Sho Kamata, Sota Sato, Jishan Wu, Hiroyuki Isobe

**Affiliations:** a Advanced Institute for Materials Research, Tohoku University, Aoba-ku, Sendai 980-8577, Japan; b JST, ERATO, Isobe Degenerate π-Integration Project, Aoba-ku, Sendai 980-8577, Japan; c Department of Chemistry, National University of Singapore, 3 Science Drive 3, Singapore 117543, Singapore; d Department of Chemistry, The University of Tokyo, Hongo, Bunkyo-ku, Tokyo 113-0033, Japan

**Keywords:** crystal structure, hepta­zethrene, substituent effect

## Abstract

The title compound, C_50_H_44_, **1**, was synthesized as a derivative of hepta­zethrene bearing two methyl and two *tert*-butyl­phenyl substituents, respectively, at the 1,9- and 7,15-positions. The asymmetric unit consists of one half of the mol­ecule, which lies about an inversion centre. Albeit remotely located, the substituents contort the hepta­zethrene plane. The *tert*-butyl­phenyl substituents stand approximately perpendicular to the core plane, with a dihedral angle of 79.09 (5)° between the phenalene ring system and the substituted benzene ring, and prevent direct inter­molecular contacts of the hepta­zethrene cores.

## Abstract 2

The title compound was synthesized as a derivative of hepta­zethrene bearing two methyl and two *tert*-butyl­phenyl substituents, respectively, at the 1,9- and 7,15-positions. Albeit remotely located, the substituents contort the hepta­zethrene plane. The phenyl substituents stand approximately perpendicular to the core plane and prevent direct inter­molecular contacts of the hepta­zethrene cores.

## Chemical context   

Heptazethrene is a polycyclic aromatic hydro­carbon with a characteristic Z-shaped mol­ecular structure. A series of hepta­zethrene derivatives have been synthesized by one of the authors, and a derivative, **2**, with methyl and silylethynyl substituents at the 1,9- and 7,15-positions has been reported as the first closed-shell congener (Li *et al.*, 2012 ▸). In the crystal structure of **2**, we noticed that the silylethynyl substituents are distorted into a non-linear geometry. Considering that the distorted structure originated from steric inter­actions between the 1,9- and 7,15-positions, we investigated substituent effects on the mol­ecular structure. Replacing the silylethynyl groups with *tert*-butyl­phenyl groups, we designed the title compound, **1**, and synthesized it *via* a route recently established for other hepta­zethrene derivatives (Hu *et al.*, 2016 ▸).
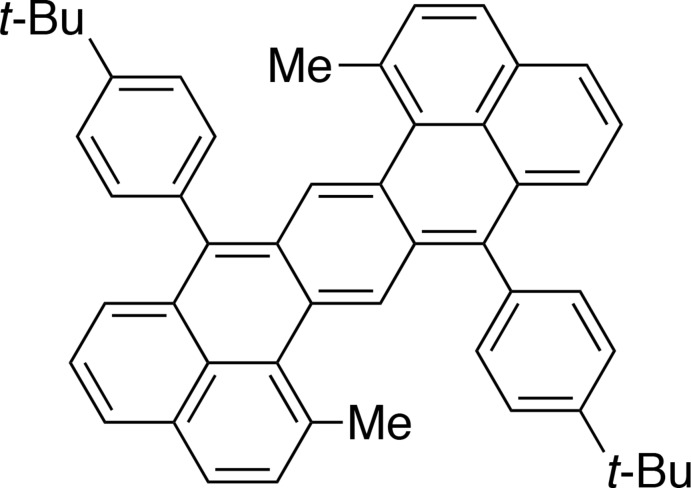


## Structural commentary   

**Figure 1:**
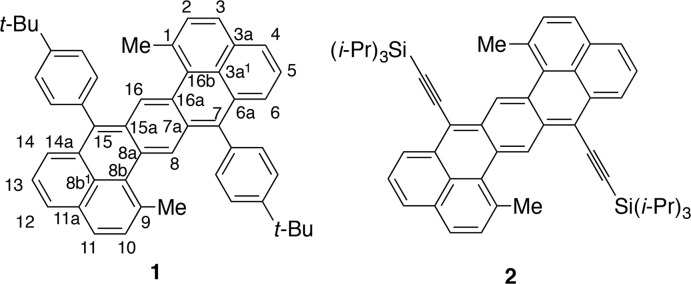
Chemical structures of hepta­zethrene derivatives. Numbers of positions are displayed for **1**.

**Figure 2:**
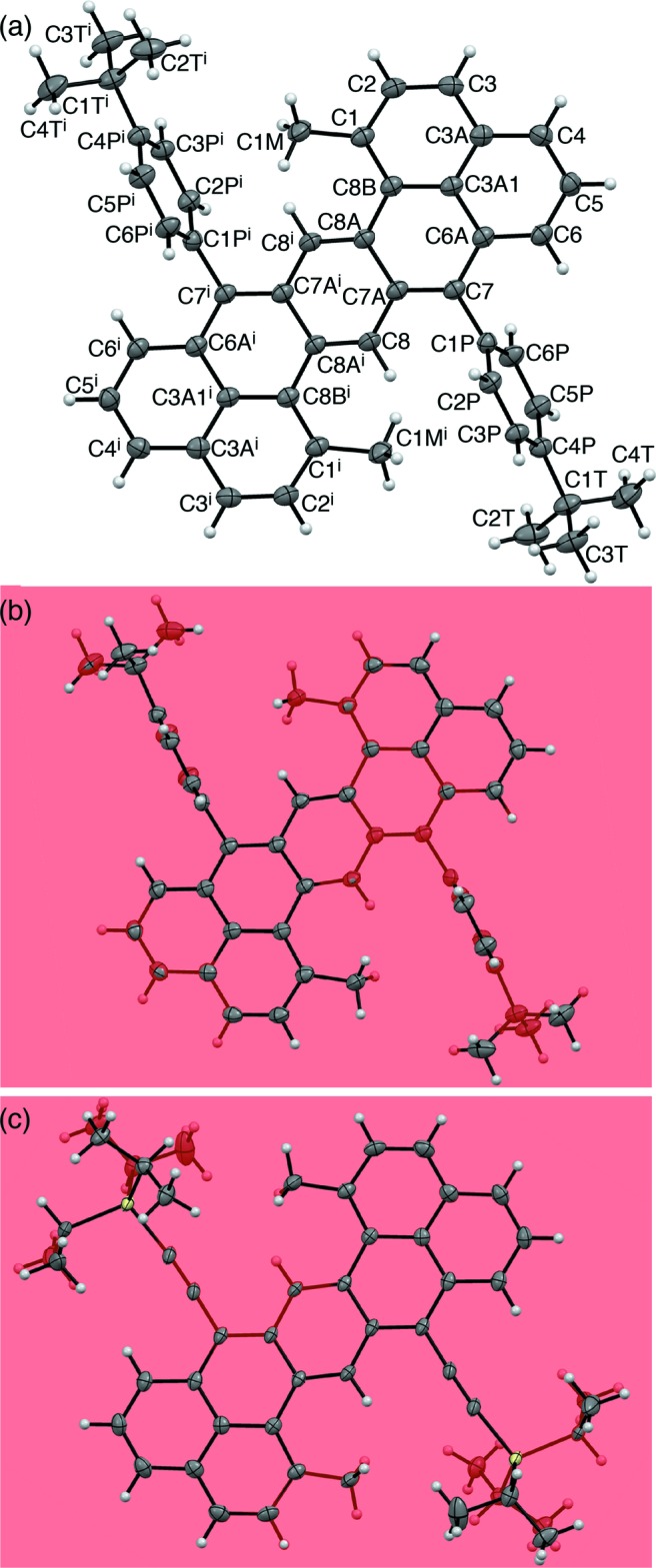
The mol­ecular structures of hepta­zethrene derivatives. Displacement ellipsoids are drawn at the 50% probability level. (*a*) **1** with the atom-numbering scheme [symmetry code: (i) 1 − *x*, 1 − *y*, 1 − *z*]. (*b*) **1** with a mean plane of 28 C atoms, viewed perpendicular to the plane shown in red. (*c*) **2** with a mean plane of 28 C atoms, viewed perpendicular to the plane shown in red.

The mol­ecular structure of **1** (Fig. 1 ▸) consists of a hepta­zethrene unit at the core, two methyl substituents at the 1,9-positions and two *tert*-butyl­phenyl substituents at the 7,15-positions. One-half of the mol­ecule is generated by the symmetry operation (1 − *x*, 1 − *y*, 1 − *z*), and carbon atoms at the 1/9-, 7/15- or 8/16-positions, for instance, are symmetrically equivalent (Fig. 2 ▸ *a*). As is the case with **2**, a typical bond-length alternation in the central hexa­gon is observed, indicating a quinoidal character for **1** (Li *et al.*, 2012 ▸). Unlike **2**, however, the hepta­zethrene core of **1** is not flat but contorted. The mean plane of the hepta­zethrene core is generated by adopting 28 carbon atoms of the core (*OLEX2*; Dolomanov *et al.*, 2009 ▸) and the deviation of the atoms from the mean plane is visualized in Fig. 2 ▸ *b*. The maximum deviation of 0.2969 (10) Å is recorded (by using *OLEX2*) for the carbon atoms at the 7- and 15-positions. The same analysis was applied to **2** (Fig. 2 ▸ *c*), and the maximum distance from the mean plane is 0.103 (3) Å for the carbon atoms at the 8- and 16-positions. The contorted structure of **1** is also evidenced by the torsion angle at the 1–16b–16a–16 (see Fig. 1 ▸) positions, is −16.91 (19)°. For **2**, the torsion angle at the same position is 5.8 (3)°, which indicates that steric inter­actions between the 1-methyl and 7-phenyl groups may result in the contorted structure.

## Supra­molecular features   

**Figure 3:**
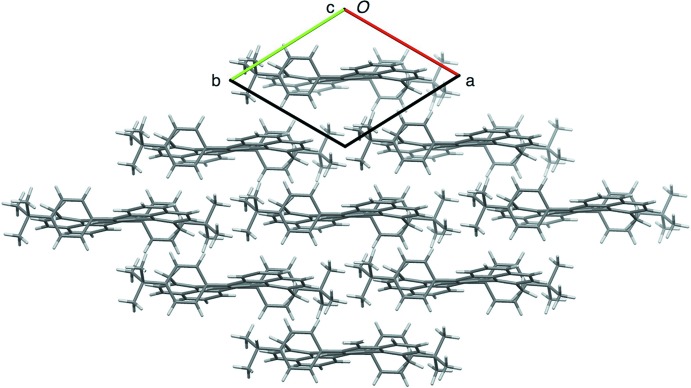
Packing diagram of **1**, viewed along the *c* axis.

As is the case of **2** (Li *et al.*, 2012 ▸), the mol­ecules of **1** form layers with the hepta­zethrene cores assembled in a parallel manner (Fig. 3 ▸). However, due to the bulky phenyl groups at the 7,15-positions, the hepta­zethrene cores do not directly contact each other. C—H⋯π inter­actions are instead observed between the hepta­zethrene core and the phenyl substituent (Table 1 ▸).

## Database survey   

A search of the Cambridge Structural Database (version 5.37 Update 2; Groom *et al.*, 2016 ▸) for hepta­zethrene derivatives returns one result, compound **2** (Li *et al.*, 2012 ▸). Two newer derivatives, 1,9-bis­(hex­yloxy)-7,15-dimesityl-hepta­zethrene and 1,9-bis­(hex­yloxy)-7,15-bis­(penta­fluoro­phen­yl)-hepta­zethrene, have recently been reported (Hu *et al.*, 2016 ▸). Detailed comparisons with compound **2** are described above. The other two derivatives possessing 7,15-phenyl groups and 1,9-alk­oxy substituents are also contorted. Two crystallographically independent mol­ecules are observed in 1,9-bis­(hex­yloxy)-7,15-dimesityl-hepta­zethrene, and the 1–16b–16a–16 torsion angles are 2.55 (19) and 13.94 (19)°. One mol­ecule is observed in 1,9-bis­(hex­yloxy)-7,15-bis­(penta­fluoro­phen­yl)-hepta­zethrene, and the corresponding torsion angle is 6.44 (18)°.

## Synthesis and crystallization   

**Figure 4:**
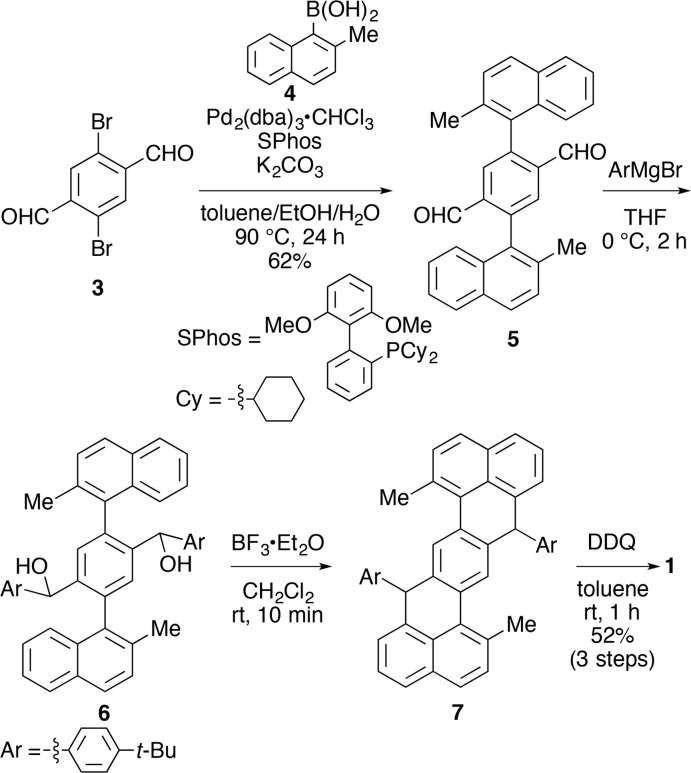
Synthesis of the title compound, **1**.

The title compound **1** was synthesized by a method reported in a literature (Hu *et al.*, 2016 ▸) with different starting materials for the introduction of different substituents (Fig. 4 ▸). A mixture of 2,5-di­bromo-terephthalaldehyde **3** (2.30 g, 7.89 mmol), 2-methyl­naphthyl­boronic acid **4** (4.41 g, 23.7 mmol), Pd_2_(dba)_3_·CHCl_3_ (407 mg, 0.393 mmol), SPhos (648 mg, 1.58 mmol) and K_2_CO_3_ (5.46 g, 39.4 mmol) was stirred in a deaerated solvent composed of toluene (70.8 ml), ethanol (17.2 ml) and water (19.0 ml) at 363 K for 24 h. The reaction was quenched by addition of saturated aqueous NH_4_Cl (50 ml). Organic materials were extracted with CHCl_3_ (30 ml × 4), and the combined organic phase was washed with brine, dried over MgSO_4_ and concentrated *in vacuo*. Crude materials were purified by silica gel column chromatography (eluent: 30% CHCl_3_/hexa­ne) to afford the coupling product **5** in 2.02 g (4.87 mmol, 62% yield) as a yellow powder. The compound **5** (1.64 g, 3.97 mmol) was dissolved in THF (80.0 mL), and to the solution was added 4-*tert*-butyl­phenyl­magnesium bromide (30.0 ml, 0.66 *M* in diethyl ether, 19.8 mmol) at 273 K. The mixture was stirred for 2 h, and saturated aqueous NH_4_Cl (20 ml) was added. Organic materials were extracted with ethyl acetate (50 ml × 3), and the combined organic phase was washed with brine, dried over Na_2_SO_4_ and concentrated *in vacuo* to give a yellow oil containing diol **6**. Without purification, the crude material was dissolved in CH_2_Cl_2_ (200 ml), and BF_3_·Et_2_O (5.10 ml, 39.5 mmol) was added at ambient temperature. After 10 min, methanol (10 ml) was added, and volatile materials were removed *in vacuo*. The crude material was washed with methanol (50 ml), and a purple solid containing the cyclized compound **7** was obtained. Without purification, the crude material was dissolved in toluene (400 ml), and to the solution was added a solution of 2,3-di­chloro-5,6-di­cyano-*para*-benzo­quinone (DDQ; 70.0 ml, 79.5 m*M* in toluene, 5.57 mmol) at ambient temperature. After 1 h, the mixture was poured onto a pad of silica gel (250 g) and eluted with toluene to afford the title compound **1**. A small amount of contaminants was noted and was removed by washing the compound with methanol (50 ml) to afford the title compound **1** (1.33 g, 2.06 mmol, 52% in three steps from **5**) as a purple solid. Suitable single crystals were grown from slow liquid–liquid diffusion of aceto­nitrile into a toluene solution of **1**.

Physical data: m.p. *ca* 643 K (decomposed); IR (ATR, neat): 567, 587, 764, 803, 825, 837, 1017, 1108, 1269, 1362, 1447, 1461, 2864, 2901, 2951 cm^−1^; ^1^H NMR (400 MHz, C_6_D_6_) *δ* 1.32 (*s*, 18H), 2.36 (*s*, 6H), 7.03 (*t*, *J* = 8.0Hz, 2H), 7.09 (*d*, *J* = 8.4 Hz, 2H), 7.25 (*d*, *J* = 7.8 Hz, 2H), 7.28 (*dt*, *J* = 8.4 Hz, 2.0 Hz, 4H), 7.36 (*d*, *J* = 8.0 Hz, 2H), 7.37 (*dt*, *J* = 8.4 Hz, 2.0 Hz, 4H), 7.38 (*d*, *J* = 8.4 Hz, 2.0 Hz, 4H), 7.72 (*s*, 2H); ^13^C NMR (100 MHz, C_6_D_6_) *δ* 26.0 (CH_3_), 31.5 (CH_3_), 34.6, 125.6 (CH), 126.1* (CH), 127.1 (CH), 127.4 (CH), 129.2 (CH), 129.7, 130.3, 131.0* (CH), 131.7, 132.6 (CH), 133.2, 133.6, 134.7, 136.0, 136.7, 150.3 (Resonances with * appeared with twofold intensities and should contain two overlapping resonances.); HR–MS (DART–TOF, positive) calculated for C_50_H_44_ [*M*+H]^+^ 645.3521, found 645.3545.

## Refinement   

Crystal data, data collection and structure refinement details are summarized in Table 2 ▸. H atoms were positioned geometrically and refined as riding, allowing for rotation of the methyl group, with *U*_iso_(H) = 1.5*U*_eq_(C) for methyl H atoms and 1.2*U*_eq_(C) for aromatic H atoms.

## Supplementary Material

Crystal structure: contains datablock(s) global, I. DOI: 10.1107/S2056989016020247/is5468sup1.cif

Structure factors: contains datablock(s) I. DOI: 10.1107/S2056989016020247/is5468Isup2.hkl

Supporting information file. DOI: 10.1107/S2056989016020247/is5468Isup3.mol

CCDC reference: 1523781

Additional supporting information:  crystallographic information; 3D view; checkCIF report

## Figures and Tables

**Table 1:** Hydrogen-bond geometry (Å, °) *Cg*1 and *Cg*2 are the centroids of the C1–C3/C3*A*/C3*A*1/C8*B* and C3*A*1/C3*A*/C4–C6/C6*A* rings, respectively.

*D*—H⋯*A*	*D*—H	H⋯*A*	*D*⋯*A*	*D*—H⋯*A*
C3*P*—H3*P*⋯*Cg*1^i^	0.95	2.80	3.6278 (15)	147
C3*T*—H3*T*2⋯*Cg*2^i^	0.98	2.97	3.7929 (17)	143

Symmetry code: (i) 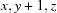.

**Table 2:** Experimental details

Crystal data	
Chemical formula	C_50_H_44_
*M*_r_	644.90
Crystal system, space group	Triclinic, *P* 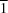
Temperature (K)	93
*a*, *b*, *c* (Å)	8.7644 (2), 9.2002 (3), 13.1212 (3)
α, β, γ (°)	105.874 (2), 95.080 (2), 115.249 (3)
*V* (Å^3^)	894.47 (5)
*Z*	1
Radiation type	Cu *K*α
μ (mm^−1^)	0.51
Crystal size (mm)	0.15 × 0.08 × 0.03

Data collection	
Diffractometer	Rigaku XtaLAB P200
Absorption correction	Multi-scan (*CrysAlis PRO*; Rigaku Oxford Diffraction, 2015 ▸)
*T*_min_, *T*_max_	0.878, 0.985
No. of measured, independent and observed [*F* ^2^ > 2.0σ(*F* ^2^)] reflections	22866, 3256, 2930
*R*_int_	0.026
(sin θ/λ)_max_ (Å^−1^)	0.602

Refinement	
*R*[*F* ^2^ > 2σ(*F* ^2^)], *wR*(*F* ^2^), *S*	0.044, 0.131, 1.08
No. of reflections	3256
No. of parameters	230
H-atom treatment	H-atom parameters constrained
Δρ_max_, Δρ_min_ (e Å^−3^)	0.37, −0.23

Computer programs: *CrysAlis PRO* (Rigaku Oxford Diffraction, 2015 ▸), *SHELXT* (Sheldrick, 2015*a* ▸), *SHELXL2016* (Sheldrick, 2015*b* ▸), *CrystalStructure* (Rigaku, 2016 ▸), *Mercury* (Macrae *et al.*, 2008 ▸), *OLEX2* (Dolomanov *et al.*, 2009 ▸) and *publCIF* (Westrip, 2010 ▸).

## supplementary crystallographic information

### Crystal data

**Table:**

C_50_H_44_	*Z* = 1
*M**_r_* = 644.90	*F*(000) = 344.00
Triclinic, *P*1	*D*_x_ = 1.197 Mg m^−^^3^
*a* = 8.7644 (2) Å	Cu *K*α radiation, λ = 1.54187 Å
*b* = 9.2002 (3) Å	Cell parameters from 15939 reflections
*c* = 13.1212 (3) Å	θ = 3.6–68.2°
α = 105.874 (2)°	µ = 0.51 mm^−^^1^
β = 95.080 (2)°	*T* = 93 K
γ = 115.249 (3)°	Block, purple
*V* = 894.47 (5) Å^3^	0.15 × 0.08 × 0.03 mm

### Data collection

**Table:**

Rigaku XtaLAB P200 diffractometer	2930 reflections with *F*^2^ > 2.0σ(*F*^2^)
Detector resolution: 5.811 pixels mm^-1^	*R*_int_ = 0.026
ω scans	θ_max_ = 68.3°, θ_min_ = 3.6°
Absorption correction: multi-scan (CrysAlis PRO; Rigaku Oxford Diffraction, 2015)	*h* = −10→10
*T*_min_ = 0.878, *T*_max_ = 0.985	*k* = −9→10
22866 measured reflections	*l* = −15→15
3256 independent reflections	

### Refinement

**Table:**

Refinement on *F*^2^	Secondary atom site location: difference Fourier map
*R*[*F*^2^ > 2σ(*F*^2^)] = 0.044	Hydrogen site location: inferred from neighbouring sites
*wR*(*F*^2^) = 0.131	H-atom parameters constrained
*S* = 1.08	*w* = 1/[σ^2^(*F*_o_^2^) + (0.0807*P*)^2^ + 0.1872*P*] where *P* = (*F*_o_^2^ + 2*F*_c_^2^)/3
3256 reflections	(Δ/σ)_max_ < 0.001
230 parameters	Δρ_max_ = 0.37 e Å^−^^3^
0 restraints	Δρ_min_ = −0.23 e Å^−^^3^
Primary atom site location: structure-invariant direct methods	

### Special details

**Table:**

Geometry. All esds (except the esd in the dihedral angle between two l.s. planes) are estimated using the full covariance matrix. The cell esds are taken into account individually in the estimation of esds in distances, angles and torsion angles; correlations between esds in cell parameters are only used when they are defined by crystal symmetry. An approximate (isotropic) treatment of cell esds is used for estimating esds involving l.s. planes.
Refinement. Refinement was performed using all reflections. The weighted R-factor (wR) and goodness of fit (S) are based on F^2^. R-factor (gt) are based on F. The threshold expression of F^2^ > 2.0 sigma(F^2^) is used only for calculating R-factor (gt).

### Fractional atomic coordinates and isotropic or equivalent isotropic displacement parameters (Å^2^)

**Table:**

	*x*	*y*	*z*	*U*_iso_*/*U*_eq_	
C1	0.77143 (15)	0.27792 (16)	0.37967 (10)	0.0282 (3)	
C2	0.82438 (16)	0.19027 (16)	0.29671 (11)	0.0304 (3)	
H2	0.901044	0.149216	0.316696	0.036*	
C3	0.77086 (16)	0.16138 (16)	0.18882 (11)	0.0294 (3)	
H3	0.802444	0.093561	0.135216	0.035*	
C3A	0.66842 (15)	0.23267 (15)	0.15725 (10)	0.0270 (3)	
C3A1	0.61706 (14)	0.32728 (14)	0.23942 (10)	0.0247 (3)	
C4	0.61937 (16)	0.21352 (16)	0.04656 (10)	0.0299 (3)	
H4	0.651358	0.147501	−0.007973	0.036*	
C5	0.52627 (16)	0.28945 (16)	0.01768 (10)	0.0308 (3)	
H5	0.492798	0.274895	−0.056770	0.037*	
C6	0.48038 (16)	0.38832 (16)	0.09751 (10)	0.0284 (3)	
H6	0.418515	0.442753	0.076400	0.034*	
C6A	0.52289 (15)	0.40922 (15)	0.20719 (10)	0.0254 (3)	
C7	0.47383 (15)	0.51085 (15)	0.28851 (10)	0.0253 (3)	
C7A	0.49880 (15)	0.51364 (15)	0.39461 (10)	0.0255 (3)	
C8	0.42937 (15)	0.59605 (15)	0.47208 (10)	0.0261 (3)	
H8	0.386896	0.666232	0.452300	0.031*	
C8A	0.57990 (15)	0.41936 (15)	0.42770 (10)	0.0249 (3)	
C8B	0.65887 (15)	0.34037 (15)	0.35078 (10)	0.0255 (3)	
C1M	0.84692 (17)	0.29973 (19)	0.49464 (11)	0.0359 (3)	
H1M1	0.953456	0.288760	0.496403	0.054*	
H1M2	0.762462	0.211359	0.517639	0.054*	
H1M3	0.873653	0.413186	0.544212	0.054*	
C1P	0.37768 (15)	0.59823 (15)	0.25632 (9)	0.0257 (3)	
C2P	0.45677 (15)	0.77354 (16)	0.27639 (10)	0.0275 (3)	
H2P	0.578450	0.840095	0.305958	0.033*	
C3P	0.36053 (16)	0.85288 (16)	0.25386 (10)	0.0278 (3)	
H3P	0.418003	0.972795	0.268045	0.033*	
C4P	0.18176 (15)	0.76070 (15)	0.21096 (10)	0.0273 (3)	
C5P	0.10460 (16)	0.58447 (16)	0.18943 (11)	0.0329 (3)	
H5P	−0.016815	0.517320	0.158986	0.039*	
C6P	0.19987 (16)	0.50500 (16)	0.21115 (11)	0.0317 (3)	
H6P	0.142991	0.384575	0.194958	0.038*	
C1T	0.06794 (16)	0.84316 (17)	0.19069 (11)	0.0333 (3)	
C2T	−0.05461 (19)	0.8203 (2)	0.26853 (15)	0.0468 (4)	
H2T1	−0.118334	0.698859	0.259859	0.070*	
H2T2	−0.137001	0.861074	0.251474	0.070*	
H2T3	0.013342	0.886463	0.343903	0.070*	
C3T	0.17399 (17)	1.03289 (17)	0.20891 (13)	0.0401 (3)	
H3T1	0.096047	1.078314	0.191671	0.060*	
H3T2	0.255721	1.048167	0.161518	0.060*	
H3T3	0.238494	1.094359	0.285279	0.060*	
C4T	−0.04149 (19)	0.7524 (2)	0.07259 (13)	0.0465 (4)	
H4T1	−0.118581	0.631892	0.061158	0.070*	
H4T2	0.035486	0.759603	0.022257	0.070*	
H4T3	−0.111104	0.807899	0.059085	0.070*	

### Atomic displacement parameters (Å^2^)

**Table:**

	*U*^11^	*U*^22^	*U*^33^	*U*^12^	*U*^13^	*U*^23^
C1	0.0275 (6)	0.0267 (6)	0.0350 (6)	0.0137 (5)	0.0094 (5)	0.0150 (5)
C2	0.0297 (6)	0.0289 (6)	0.0402 (7)	0.0168 (5)	0.0122 (5)	0.0169 (5)
C3	0.0297 (6)	0.0253 (6)	0.0382 (7)	0.0147 (5)	0.0143 (5)	0.0130 (5)
C3A	0.0256 (6)	0.0229 (6)	0.0331 (6)	0.0092 (5)	0.0105 (5)	0.0129 (5)
C3A1	0.0226 (5)	0.0204 (6)	0.0323 (6)	0.0087 (5)	0.0088 (5)	0.0124 (5)
C4	0.0327 (6)	0.0255 (6)	0.0324 (6)	0.0129 (5)	0.0126 (5)	0.0112 (5)
C5	0.0357 (7)	0.0287 (7)	0.0286 (6)	0.0132 (5)	0.0095 (5)	0.0131 (5)
C6	0.0306 (6)	0.0263 (6)	0.0319 (6)	0.0130 (5)	0.0088 (5)	0.0153 (5)
C6A	0.0239 (6)	0.0221 (6)	0.0312 (6)	0.0088 (5)	0.0082 (5)	0.0132 (5)
C7	0.0248 (6)	0.0223 (6)	0.0309 (6)	0.0106 (5)	0.0074 (5)	0.0128 (5)
C7A	0.0256 (6)	0.0229 (6)	0.0307 (6)	0.0114 (5)	0.0077 (5)	0.0131 (5)
C8	0.0272 (6)	0.0247 (6)	0.0320 (6)	0.0145 (5)	0.0078 (5)	0.0137 (5)
C8A	0.0247 (6)	0.0224 (6)	0.0290 (6)	0.0112 (5)	0.0058 (4)	0.0110 (5)
C8B	0.0239 (6)	0.0218 (6)	0.0315 (6)	0.0093 (5)	0.0078 (5)	0.0124 (5)
C1M	0.0378 (7)	0.0472 (8)	0.0390 (7)	0.0293 (6)	0.0127 (6)	0.0218 (6)
C1P	0.0294 (6)	0.0279 (6)	0.0255 (6)	0.0152 (5)	0.0097 (5)	0.0137 (5)
C2P	0.0245 (6)	0.0276 (6)	0.0327 (6)	0.0117 (5)	0.0083 (5)	0.0144 (5)
C3P	0.0283 (6)	0.0237 (6)	0.0351 (6)	0.0123 (5)	0.0098 (5)	0.0144 (5)
C4P	0.0277 (6)	0.0269 (6)	0.0315 (6)	0.0142 (5)	0.0091 (5)	0.0135 (5)
C5P	0.0256 (6)	0.0279 (7)	0.0434 (7)	0.0104 (5)	0.0041 (5)	0.0146 (6)
C6P	0.0311 (6)	0.0229 (6)	0.0409 (7)	0.0114 (5)	0.0055 (5)	0.0139 (5)
C1T	0.0261 (6)	0.0300 (7)	0.0477 (7)	0.0144 (5)	0.0088 (5)	0.0171 (6)
C2T	0.0397 (8)	0.0401 (8)	0.0768 (11)	0.0247 (7)	0.0285 (7)	0.0292 (8)
C3T	0.0315 (7)	0.0308 (7)	0.0654 (9)	0.0172 (6)	0.0110 (6)	0.0232 (7)
C4T	0.0373 (7)	0.0427 (8)	0.0607 (9)	0.0206 (7)	−0.0017 (7)	0.0205 (7)

### Geometric parameters (Å, º)

**Table:**

C1—C2	1.4068 (18)	C1M—H1M2	0.9800
C1—C8B	1.4084 (17)	C1M—H1M3	0.9800
C1—C1M	1.5159 (17)	C1P—C2P	1.3902 (17)
C2—C3	1.3655 (18)	C1P—C6P	1.3905 (17)
C2—H2	0.9500	C2P—C3P	1.3896 (17)
C3—C3A	1.4117 (17)	C2P—H2P	0.9500
C3—H3	0.9500	C3P—C4P	1.3928 (17)
C3A—C4	1.4178 (17)	C3P—H3P	0.9500
C3A—C3A1	1.4247 (17)	C4P—C5P	1.3950 (17)
C3A1—C8B	1.4335 (17)	C4P—C1T	1.5331 (17)
C3A1—C6A	1.4394 (17)	C5P—C6P	1.3804 (17)
C4—C5	1.3689 (18)	C5P—H5P	0.9500
C4—H4	0.9500	C6P—H6P	0.9500
C5—C6	1.3970 (18)	C1T—C3T	1.5220 (18)
C5—H5	0.9500	C1T—C2T	1.5361 (19)
C6—C6A	1.3926 (17)	C1T—C4T	1.538 (2)
C6—H6	0.9500	C2T—H2T1	0.9800
C6A—C7	1.4409 (17)	C2T—H2T2	0.9800
C7—C7A	1.3813 (17)	C2T—H2T3	0.9800
C7—C1P	1.4952 (16)	C3T—H3T1	0.9800
C7A—C8	1.4353 (17)	C3T—H3T2	0.9800
C7A—C8A	1.4572 (16)	C3T—H3T3	0.9800
C8—C8A^i^	1.3664 (17)	C4T—H4T1	0.9800
C8—H8	0.9500	C4T—H4T2	0.9800
C8A—C8B	1.4756 (17)	C4T—H4T3	0.9800
C1M—H1M1	0.9800		
			
C2—C1—C8B	118.76 (11)	C1—C1M—H1M3	109.5
C2—C1—C1M	115.40 (11)	H1M1—C1M—H1M3	109.5
C8B—C1—C1M	125.82 (11)	H1M2—C1M—H1M3	109.5
C3—C2—C1	123.15 (12)	C2P—C1P—C6P	117.68 (11)
C3—C2—H2	118.4	C2P—C1P—C7	122.52 (11)
C1—C2—H2	118.4	C6P—C1P—C7	119.66 (11)
C2—C3—C3A	119.76 (12)	C3P—C2P—C1P	121.00 (11)
C2—C3—H3	120.1	C3P—C2P—H2P	119.5
C3A—C3—H3	120.1	C1P—C2P—H2P	119.5
C3—C3A—C4	121.12 (11)	C2P—C3P—C4P	121.54 (11)
C3—C3A—C3A1	118.58 (11)	C2P—C3P—H3P	119.2
C4—C3A—C3A1	120.30 (11)	C4P—C3P—H3P	119.2
C3A—C3A1—C8B	120.68 (11)	C3P—C4P—C5P	116.84 (11)
C3A—C3A1—C6A	118.01 (11)	C3P—C4P—C1T	123.67 (11)
C8B—C3A1—C6A	121.30 (11)	C5P—C4P—C1T	119.45 (11)
C5—C4—C3A	120.49 (11)	C6P—C5P—C4P	121.80 (11)
C5—C4—H4	119.8	C6P—C5P—H5P	119.1
C3A—C4—H4	119.8	C4P—C5P—H5P	119.1
C4—C5—C6	120.12 (11)	C5P—C6P—C1P	121.12 (12)
C4—C5—H5	119.9	C5P—C6P—H6P	119.4
C6—C5—H5	119.9	C1P—C6P—H6P	119.4
C6A—C6—C5	121.78 (12)	C3T—C1T—C4P	112.58 (10)
C6A—C6—H6	119.1	C3T—C1T—C2T	109.31 (12)
C5—C6—H6	119.1	C4P—C1T—C2T	108.22 (11)
C6—C6A—C3A1	119.22 (11)	C3T—C1T—C4T	108.22 (11)
C6—C6A—C7	121.14 (11)	C4P—C1T—C4T	109.63 (11)
C3A1—C6A—C7	119.64 (11)	C2T—C1T—C4T	108.82 (12)
C7A—C7—C6A	120.21 (11)	C1T—C2T—H2T1	109.5
C7A—C7—C1P	119.24 (11)	C1T—C2T—H2T2	109.5
C6A—C7—C1P	120.20 (10)	H2T1—C2T—H2T2	109.5
C7—C7A—C8	120.18 (11)	C1T—C2T—H2T3	109.5
C7—C7A—C8A	121.18 (11)	H2T1—C2T—H2T3	109.5
C8—C7A—C8A	118.35 (11)	H2T2—C2T—H2T3	109.5
C8A^i^—C8—C7A	124.58 (11)	C1T—C3T—H3T1	109.5
C8A^i^—C8—H8	117.7	C1T—C3T—H3T2	109.5
C7A—C8—H8	117.7	H3T1—C3T—H3T2	109.5
C8^i^—C8A—C7A	116.82 (11)	C1T—C3T—H3T3	109.5
C8^i^—C8A—C8B	124.06 (11)	H3T1—C3T—H3T3	109.5
C7A—C8A—C8B	119.05 (11)	H3T2—C3T—H3T3	109.5
C1—C8B—C3A1	118.48 (11)	C1T—C4T—H4T1	109.5
C1—C8B—C8A	124.42 (11)	C1T—C4T—H4T2	109.5
C3A1—C8B—C8A	117.10 (11)	H4T1—C4T—H4T2	109.5
C1—C1M—H1M1	109.5	C1T—C4T—H4T3	109.5
C1—C1M—H1M2	109.5	H4T1—C4T—H4T3	109.5
H1M1—C1M—H1M2	109.5	H4T2—C4T—H4T3	109.5
			
C8B—C1—C2—C3	−0.49 (19)	C2—C1—C8B—C3A1	−6.23 (17)
C1M—C1—C2—C3	−179.09 (12)	C1M—C1—C8B—C3A1	172.20 (11)
C1—C2—C3—C3A	5.13 (19)	C2—C1—C8B—C8A	173.80 (11)
C2—C3—C3A—C4	176.25 (11)	C1M—C1—C8B—C8A	−7.76 (19)
C2—C3—C3A—C3A1	−2.76 (17)	C3A—C3A1—C8B—C1	8.50 (17)
C3—C3A—C3A1—C8B	−4.01 (17)	C6A—C3A1—C8B—C1	−171.30 (10)
C4—C3A—C3A1—C8B	176.97 (10)	C3A—C3A1—C8B—C8A	−171.53 (10)
C3—C3A—C3A1—C6A	175.79 (10)	C6A—C3A1—C8B—C8A	8.67 (16)
C4—C3A—C3A1—C6A	−3.22 (17)	C8^i^—C8A—C8B—C1	−16.91 (19)
C3—C3A—C4—C5	−177.21 (11)	C7A—C8A—C8B—C1	166.07 (11)
C3A1—C3A—C4—C5	1.78 (18)	C8^i^—C8A—C8B—C3A1	163.12 (11)
C3A—C4—C5—C6	0.71 (18)	C7A—C8A—C8B—C3A1	−13.90 (16)
C4—C5—C6—C6A	−1.71 (19)	C7A—C7—C1P—C2P	81.89 (15)
C5—C6—C6A—C3A1	0.18 (18)	C6A—C7—C1P—C2P	−104.89 (14)
C5—C6—C6A—C7	−179.66 (11)	C7A—C7—C1P—C6P	−93.64 (14)
C3A—C3A1—C6A—C6	2.25 (16)	C6A—C7—C1P—C6P	79.58 (14)
C8B—C3A1—C6A—C6	−177.94 (10)	C6P—C1P—C2P—C3P	1.16 (17)
C3A—C3A1—C6A—C7	−177.91 (10)	C7—C1P—C2P—C3P	−174.46 (10)
C8B—C3A1—C6A—C7	1.89 (17)	C1P—C2P—C3P—C4P	0.31 (18)
C6—C6A—C7—C7A	172.31 (11)	C2P—C3P—C4P—C5P	−1.40 (18)
C3A1—C6A—C7—C7A	−7.52 (17)	C2P—C3P—C4P—C1T	176.39 (11)
C6—C6A—C7—C1P	−0.83 (17)	C3P—C4P—C5P—C6P	1.05 (19)
C3A1—C6A—C7—C1P	179.33 (10)	C1T—C4P—C5P—C6P	−176.83 (12)
C6A—C7—C7A—C8	−171.72 (10)	C4P—C5P—C6P—C1P	0.4 (2)
C1P—C7—C7A—C8	1.50 (17)	C2P—C1P—C6P—C5P	−1.51 (18)
C6A—C7—C7A—C8A	2.04 (17)	C7—C1P—C6P—C5P	174.24 (11)
C1P—C7—C7A—C8A	175.25 (10)	C3P—C4P—C1T—C3T	7.25 (18)
C7—C7A—C8—C8A^i^	168.00 (12)	C5P—C4P—C1T—C3T	−175.02 (12)
C8A—C7A—C8—C8A^i^	−5.93 (19)	C3P—C4P—C1T—C2T	−113.67 (14)
C7—C7A—C8A—C8^i^	−168.40 (12)	C5P—C4P—C1T—C2T	64.06 (15)
C8—C7A—C8A—C8^i^	5.47 (18)	C3P—C4P—C1T—C4T	127.77 (13)
C7—C7A—C8A—C8B	8.83 (17)	C5P—C4P—C1T—C4T	−54.50 (15)
C8—C7A—C8A—C8B	−177.30 (10)		

Symmetry code: (i) −*x*+1, −*y*+1, −*z*+1.

### Hydrogen-bond geometry (Å, º)

*Cg*1 and *Cg*2 are the centroids of the C1–C3/C3A/C3A1/C8B and C3A1/C3A/C4–C6/C6A rings, respectively.

**Table:**

*D*—H···*A*	*D*—H	H···*A*	*D*···*A*	*D*—H···*A*
C3*P*—H3*P*···*Cg*1^ii^	0.95	2.80	3.6278 (15)	147
C3*T*—H3*T*2···*Cg*2^ii^	0.98	2.97	3.7929 (17)	143

Symmetry code: (ii) *x*, *y*+1, *z*.
